# Current Progress In Orthopaedics

**DOI:** 10.5704/MOJ.1503.013

**Published:** 2015-03

**Authors:** Saw Aik

**Affiliations:** Department of Orthopaedic Surgery University Malaya Medical Centre, Malaysia

This book provides an overview on current progress in various orthopaedic subspecialties. It is the collaborative product of three main editors and 55 experts from different parts of the world with the support of SICOT (International Society of Orthopaedic Surgery and traumatology).

The content covers congenital, developmental and degenerative conditions, trauma, growth and infection related disorders, and clinically relevant basic scientific research. Congenital abnormalities affecting the limbs remains a great challenge to orthopaedic surgeons, but development in this field is swinging from amputation and prosthetic fitting to bone and soft tissue reconstruction due to better understanding of underlying pathology and improvement in surgical techniques. Management of congenital talipes equinovarus (CTEV) has changed dramatically favouring non-operative approach (Ponseti technique), while early surgical intervention for brachial plexus birth palsy (BPBP) is gaining popularity with better defined indications. An update on management of cerebral palsy children stresses the importance of disease categorization and single event multilevel surgery (SEMLS) approach.

A few chapters are dedicated to provide comprehensive review of various spine conditions. They include management of sagittal and coronal spine deformity, disc disease, Tuberculosis, osteoporotic fracture and metastasis.

Femoro-acetabular impingement (FAI) has recently been recognized as a source of hip pain and a cause for early osteoarthritis. Hip arthroscopy and surgical hip dislocation are some of the newly developed procedures for evaluation and treatment of this condition. In addition to updates on surgical fixation for femur neck and distal radius fractures, a chapter is assigned for review of the role of functional nonoperative treatment for humerus fracture.

There is a good review on surgical options and implant selection for degenerative disease of major joints, with special focus on younger patients and those with early osteoarthritis. Parallel to improvement in survival rate among patients with malignant musculoskeletal tumours, there has been increasing preference for limb salvage with the use of endoprosthesis or biological reconstruction. One chapter describes the basic principles of organising a screening program, and reviews the practice of screening for scoliosis and developmental dysplasia of hip. Finally, another chapter provides an update on autologous chondrocyte implantation (ACI) and non-cell-based approaches to cartilage repair.

Most of the progress described in this book are related to innovation of surgical techniques and better understanding of disease processes, rather than on improvement in implant designs or biomaterial. Although some of the recommendations may not be based on strong clinical evidence, they provide the general orthopaedic surgeons with some ideas about the direction of future advancement.

I would like to recommend this book to general orthopaedic surgeons who are interested to keep pace with the rapid development in orthopaedics. Even for those who have already subspecialized, updates by experts in the respective field will still be beneficial.

